# Human *Salmonella* and Concurrent Decreased Susceptibility to Quinolones and Extended-Spectrum Cephalosporins

**DOI:** 10.3201/eid1311.061438

**Published:** 2007-11

**Authors:** Jean M. Whichard, Kathryn Gay, Jennifer E. Stevenson, Kevin J. Joyce, Kara L. Cooper, Michael Omondi, Felicita Medalla, George A. Jacoby, Timothy J. Barrett

**Affiliations:** * Centers for Disease Control and Prevention, Atlanta, Georgia, USA; † Lahey Clinic, Burlington, Massachusetts, USA; 1 Current affiliation: Banfield, The Pet Hospital, Philadelphia, Pennsylvania, USA; 2 Current affiliation: Washington State University, Pullman, Washington, USA

**Keywords:** Drug resistance, microbial, microbial sensititivity tests, quinolones, Salmonella enterica, cephalosporins, beta-lactamases, research

## Abstract

The National Antimicrobial Resistance Monitoring System monitors susceptibility among Enterobacteriaceae in humans in the United States. We studied isolates exhibiting decreased susceptibility to quinolones (nalidixic acid MIC >32 µg/mL or ciprofloxacin MIC >0.12 µg/mL) and extended-spectrum cephalosporins (ceftiofur or ceftriaxone MIC >2 µg/mL) during 1996–2004. Of non-Typhi *Salmonella,* 0.19% (27/14,043) met these criteria: 11 Senftenberg; 6 Typhimurium; 3 Newport; 2 Enteridis; and 1 each Agona, Haifa, Mbandaka, Saintpaul, and Uganda. Twenty-six isolates had *gyrA* mutations (11 at codon 83 only, 3 at codon 87 only, 12 at both). All Senftenberg isolates had *parC* mutations (S80I and T57S); 6 others had the T57S mutation. The Mbandaka isolate contained *qnrB2*. Eight isolates contained *bla*_CMY-2_; 1 Senftenberg contained *bla*_CMY-23_. One Senftenberg and 1Typhimurium isolate contained *bla*_SHV-12_; the Mbandaka isolate contained *bla*_SHV-30_. Nine Senftenberg isolates contained *bla*_OXA-1_; 1 contained *bla*_OXA-9_. Further studies should address patient outcomes, risk factors, and resistance dissemination prevention strategies.

## Abstract 2

For complicated infections, decreased susceptibility could compromise treatment with drugs from either antimicrobial class.

Although antimicrobial agents are not indicated for uncomplicated *Salmonella* infections, fluoroquinolones and extended-spectrum cephalosporins are potentially life-saving treatments for extraintestinal infections (*1*). The National Antimicrobial Resistance Monitoring System (NARMS) has monitored antimicrobial drug resistance among enteric pathogens since 1996. NARMS has documented decreased susceptibility to each of these drug classes, in most instances among separate serotypes (*2*). Historically, decreased susceptibility to fluoroquinolones, which can be monitored by tracking resistance to nalidixic acid, has been noted among *Salmonella* serotypes (ser.) Typhi, Senftenberg, and Virchow (*2*,*3*). More recently, decreased susceptibility to fluoroquinolones has been noted among *Salmonella* ser. Enteritidis (*4*). Decreased fluoroquinolone susceptibility has also been seen among nalidixic acid–susceptible isolates (*5*). Extended-spectrum cephalosporin resistance was noted among 15 non-Typhi *Salmonella* NARMS isolates (including 12 ser. Typhimurium) during 1996–1998 (*6*). In all instances, extended-spectrum cephalosporin resistance was the result of *bla*_CMY-2_, a class C plasmid-encoded *ampC* gene (*7*). In addition to conferring resistance or decreased susceptibility to extended-spectrum cephalosporins such as ceftiofur and ceftriaxone, this gene also confers resistance to ampicillin (AMP), amoxillin-clavulanate, cephalothin, and cefoxitin. This AmpC resistance phenotype has been seen in strains of *Salmonella* ser. Newport along with resistance to other drugs including chloramphenicol, streptomycin, sulfamethoxazole, and tetracycline. This MDRAmpC strain rose from 1% (1/77) of *Salmonella* ser. Newport submissions in 1998 to 25% (31/124) in 2001 (*4*). CMY β-lactamases are largely responsible for extended-spectrum cephalosporin resistance among *Salmonella* ser. Newport, Typhimurium, and others isolated in North America (*6*,*8*).

Coresistance to fluoroquinolones and extended-spectrum cephalosporins would limit therapeutic options for *Salmonella* infections. Decreased susceptibility to both drug classes was identified in Thailand in 1993 (ser. Anatum, Derby, Enteritidis, Typhimurium, Weltevreden, and I 4,5,12:i:-) (*9*), the United Kingdom in 1998 (ser. Senftenberg, Typhimurium, and Virchow) (*10*), Belgium as early as 2001 (ser. Virchow) (*11*), India in 2002 (ser. Typhi) (*12*), the United States in 2002 (ser. Mbandaka) (*13*), France in 2003 (*11*), and Taiwan in 2004 (ser. Choleraesuis, Cairo, and Kaduna) (*14*). In the United States, 27 (4.6%) of 588 *Salmonella* ser*.* Typhimurium isolates (clinical and slaughter) obtained from food animals in 1999 were resistant to ceftiofur and nalidixic acid: 22 (81%) from turkeys, 4 (15%) from horses, and 1 (4%) from cattle (*15*).

To understand coresistance to both antimicrobial classes among *Salmonella* isolates obtained from humans in the United States, we studied the NARMS human collection from 1996 through 2004, looking for decreased susceptibility to quinolones and extended-spectrum cephalosporins. Information for some of the isolates has been presented elsewhere (*3*,*13*,*16*–*18*). We present the molecular epidemiology of this phenotype and mechanisms responsible for its decreased susceptibility.

## Materials and Methods

### Isolates and Antimicrobial Drug Susceptibility Testing

NARMS-participating state and local public health laboratories submitted non-Typhi *Salmonella* isolates to the Centers for Disease Control and Prevention (CDC) for antimicrobial susceptibility testing: every 10th isolate from 1996 through 2002 and every 20th isolate from 2003 to present. Serotypes were determined by the submitting laboratory and, for this study, were confirmed by the CDC National *Salmonella* Reference Laboratory according to the Kaufmann-White scheme as described (*19*). MICs were determined by using broth microdilution (Sensititre, Westlake, OH, USA). Isolates exhibiting an amikacin MIC >4 μg/mL were confirmed by Etest (ABBiodisk, Piscataway, NJ, USA). Criteria for decreased susceptibility to quinolones and extended-spectrum cephalosporins were as follows: MIC >32 μg/mL for nalidixic acid or >0.12 μg/mL for ciprofloxacin and >2 μg/mL for ceftiofur or ceftriaxone. Susceptibility testing was performed according to manufacturer’s instructions by using control strains *Escherichia coli* ATCC25922 and ATCC35218, and *Klebsiella pneumoniae* ATCC700603 (for extended-spectrum β-lactamase [ESBL] confirmation only). When available, Clinical Laboratory Standards Institute (CLSI) guidelines were used for interpretation (*20*).

### Isoelectric Focusing for β-Lactamases

The methods of Rasheed et al. were used with modification (*21*). Three-hour trypticase soy broth cultures (grown at 37°C with shaking at 300 rpm) were pelleted, resuspended in 0.2% sodium acetate to 5% of original culture volume, and freeze-thawed 4 times (dry ice/ethanol bath and 37°C water bath). Preparations were diluted 2-fold with distilled water and swirled occasionally on ice for 30 min. Supernatants were collected after centrifugation (30 min at 20,200× *g*), and 3–5-µL aliquots were resolved for 1.5 h on Ampholine PAGplate polyacrylamide gels, pH 3.5–9.5 (APBiotech**,** Piscataway, NJ, USA). Gels were stained with nitrocefin (500 μg/mL; Becton Dickinson, Franklin Lakes, NJ, USA). Isoelectric points (pIs) were estimated by comparison with the following standard β-lactamases: TEM-12 (pI 5.25), KPC-2 (pI 6.7), SHV-3 (pI 7.0), SHV-18 (pI 7.8), and MIR-1 (pI 8.4).

### PCR Detection of Antimicrobial Drug Resistance Genes

Presence of *qnr* genes was determined by using PCR with primers QP1 and QP2 for *qnrA* (*22*), FQ1 and FQ2 for *qnrB* (*23*), and 5′-ATGGAAACCTACAATCATAC-3′ and 5′-AAAAACACCTCGACTTAAGT-3′ for *qnrS*. The *qnrB* allele was determined by amplification and sequencing with primers FQ1 and FQ2. Screening for *aac(6′)-Ib-cr* was performed as described (*24*). Primer pairs used for amplification of β-lactamase genes were: *bla*_CMY_ (5′-ATGATGAAAAAATCGTTATGC-3′) and (5′-TTGCAGCTTTTCAAGAATGCGC-3′) (*25*); *bla*_OXA-1_ (5′-AATGGCACCAGATTCAACTT-3′) and 5′-CTTGGCTTTTATGCTTGATG-3′) (*26*); *bla*_TEM_ (5′-TTCTTGAAGACGAAAGGGC-3′) and (5′-ACGCTCAGTGGAACGAAAAC-3′) (*27*); and *bla*_SHV_ (5′-GGTTATGCGTTATATTCGCC-3′) and (5′-TTAGCGTTGCCAGTGCTC-3′) (*28*) or at Lahey (5′-GCCGGGTTATTCTTATTTGTC-3′) and (5′-TCTTTCCGATGCCGCCGCCAG-3′) (*29*). *bla*_CTX-M_ genes were screened by using a multiplex PCR assay (*30*).

### DNA Sequencing

Full-length sequences were obtained for β-lactamase genes. A 255-bp region covering the quinolone-resistant determining region (QRDR) of *gyrA* (Met52 to Leu137) was amplified by using primers gyrA1: 5′-CATGAACGTATTGGGCAATG-3′ and gyrA2: 5′-AGATCGGCCATCAGTTCGTG-3′. QRDRs of *gyrB*, *parC*, and *parE* were amplified and sequenced by using previously described primers (*31*), except primers parCF (5′-ATCGTGCGTTGCCGTTTAT-3′) and parCR (5′-GCCGCTTTCGCCACTTC-3′) were used to enhance coverage of *parC*. Amplicons were sequenced by using ABI Big-Dye 3.1 chemistry and ABI 3730XL automated DNA sequencers (PE Biosystems, Foster City, CA, USA). Analysis was performed by using BioEdit (www.mbio.ncsu.edu/BioEdit/bioedit.htmL) or SeqMan software (DNAStar, Madison, WI, USA). QRDR sequences of *gyrA*, *gyrB*, *parC*, and *parE* were compared with those of *Salmonella* ser*.* Typhimurium LT2 (GenBank accession nos. AE008801, AE008878, AE008846, and AE008846, respectively).

### Pulsed-Field Gel Electrophoresis (PFGE)

PFGE was performed as previously described (*32*). Isolates that produced indistinguishable patterns with *Xba*I (Roche Molecular Biochemicals, Indianapolis, IN, USA) were restricted with *Bln*I. Patterns were analyzed by using the BioNumerics version 4.0 software (Applied Maths, Sint-Martens-Latem, Belgium) and compared by unweighted pair group method with averages by using the Dice coefficient with a 1.5% band position tolerance window. The DNA sequence and deduced amino acid sequence for the *Salmonella* ser. Senftenberg *bla*_CMY-23_ gene were assigned GenBank accession no. DQ463751.

## Results

Decreased susceptibility to quinolones and extended-spectrum cephalosporins was first noted in NARMS data in 1997 and represented 0.19% (27/14,043) of non-Typhi *Salmonella* from 1996 through 2004 (Table 1). *Salmonella* ser. Senftenberg was the most frequent serotype (n = 11), followed by Typhimurium (n = 6), Newport (n = 3), and Enteritidis (n = 2). The phenotype was found in 9 different serotypes (Table 2).

**Table 1:** NARMS non-Typhi *Salmonella* serotypes with decreased susceptibility to quinolones and extended-spectrum cephalosporins, United States, 1996–2004*

Year	No. that met MIC criteria/total tested (%)	Serotype				
		Senftenberg	Typhimurium	Newport	Enteritidis	Other (no.)
1996	0/1,324 (0)					
1997	1/1,301 (0.08)		1			
1998	1/1,460 (0.07)			1		
1999	1/1,497 (0.07)	1				
2000	4/1,377 (0.29)	2	1		1	
2001	4/1,419 (0.28)	2	1			Haifa (1)
2002	5/2,008 (0.25)	1	2	1		Mbandaka (1)
2003	4/1,864 (0.21)	2			1	Agona (1)
2004	7/1,793 (0.39)	3	1	1		Saintpaul (1), Uganda (1)
Total	27/14,043 (0.19)	11	6	3	2	5

*NARMS, National Antimicrobial Resistance Monitoring System. Reduced susceptibility to quinolones and extended-spectrum cephalosporins defined as MIC >32 μg/mL for nalidixic acid or >0.12 μg/mL for ciprofloxacin and >2 μg/mL for ceftiofur or >2 μg/mL for ceftriaxone.

**Table 2:** Isolate, year reported, state, and serotype for NARMS non-Typhi *Salmonella* isolates with decreased susceptibility to quinolones and extended-spectrum cephalosporins, United States, 1996–2004*

Isolate	Year	State	Serotype
AM18280	2003	TX	Agona
AM09124	2000	CA	Enteritidis
AM15266	2003	IL	Enteritidis
AM12389	2001	NJ	Haifa
AM15010	2002	NY	Mbandaka
AM03005	1998	NY	Newport
AM15201	2002	ME	Newport
AM21465	2004	GA	Newport
AM20428	2004	GA	Saintpaul
AM06960	1999	FL	Senftenberg
AM08081	2000	FL	Senftenberg
AM08208	2000	GA	Senftenberg
AM09864	2001	FL	Senftenberg
AM11007	2001	MA	Senftenberg
AM14058	2002	TX	Senftenberg
AM16094	2003	FL	Senftenberg
AM18622	2003	FL	Senftenberg
AM19422	2004	FL	Senftenberg
AM20227	2004	GA	Senftenberg
AM20256	2004	FL	Senftenberg
AM02544	1997	MN	Typhimurium
AM08739	2000	KS	Typhimurium
AM11682	2001	NY	Typhimurium
AM14364	2002	WI	Typhimurium
AM14807	2002	NY	Typhimurium
AM20205	2004	PA	Typhimurium
AM19537	2004	CA	Uganda

*NARMS, National Antimicrobial Resistance Monitoring System.

PFGE comparison by *Xba*I and, if applicable, *Bln*I restriction showed that 15/27 *Salmonella* isolates differed by >1 band. No indistinguishable patterns among different *Salmonella* serotypes were identified. Of the 3 ser. Newport isolates tested, 2 (AM15201 and AM21465) had indistinguishable *Xba*I patterns but different *Bln*I patterns (87.51% similarity). The 2 Enteritidis isolates (AM09124 and AM15266) were indistinguishable by both enzymes. Of the 11 ser. Senftenberg isolates, 5 exhibited unique *Xba*I PFGE patterns, while the remaining 6 were separated into 2 groups with indistinguishable *Xba*I PFGE patterns (group 1: AM06960, AM08081, AM16094, and AM19422; group 2: AM20227 and AM20256). *Bln*I restriction demonstrated that AM19422 differed from the other group 1 isolates by a single band difference (97.44% similarity). PFGE results for some of the Senftenberg isolates are described elsewhere (*16*,*18*). All Typhimurium isolates exhibited unique *Xba*I PFGE patterns (77%–93% similarity).

Antimicrobial drug susceptibility results are presented in Table 3. For nalidixic acid, 25 isolates exhibited an MIC >32 μg/mL, and 2 (Mbandaka and Newport) had an MIC of 16 μg/mL. For ciprofloxacin, MICs of 0.12–0.5 μg/mL were found for all isolates except the 11 Senftenberg, for which the MIC was >4 μg/mL. For ceftiofur, 14 isolates exhibited resistance (MIC >8 μg/mL). For ceftriaxone, 2 isolates exhibited resistance according to the current CLSI breakpoint (64 μg/mL), and 7 exhibited intermediate resistance (MIC 16 or 32 μg/mL). Three isolates (Mbandaka, Senftenberg, and Typhimurium) exhibited an ESBL phenotype according to ceftazidime and cefotaxime MIC alone and with clavulanate. Seven isolates exhibited the MDRAmpC phenotype, including 1 Agona, 2 Newport, 3 Typhimurium, and 1 Uganda. According to current CLSI guidelines, 1 isolate (ser. Senftenberg) was fully resistant to ciprofloxacin, ceftriaxone, ceftazidime, and cefotaxime.

**Table 3:** Susceptibility results for NARMS non-Typhi *Salmonella* isolates with decreased susceptibility to quinolones and extended-spectrum cephalosporins, United States, 1996–2004*

Isolate	Resistance								
	NAL	CIP	XNL	CRO	TAZ	TAZ/ CLAV	FOT	FOT/ CLAV	Other†
AM18280	>32	0.25	>8	16	32	16/4	16	8/4	AMP, AMC, CHL, FOX, KAN, STR, SUL, SXT, TET
AM09124	>32	0.5	2	<0.25	0.5	0.25/4	0.25	0.12/4	ND
AM15266	>32	0.5	2	<0.25	0.5	0.25/4	0.25	0.12/4	(CHL)
AM12389	>32	0.5	2	<0.25	0.25	0.25/4	0.1	0.12/4	(CHL), SUL, SXT, TET
AM15010	16	0.25	8	8	64	0.5/4	4	0.25/4	AMP, (CHL), SUL, SXT
AM03005	16	0.25	2	0.5	0.25	0.12/4	0.12	≤0.06/4	AMP, AMC, CHL, FOX, (GEN), KAN, STR, SUL, SXT
AM15201	>32	0.12	>8	8	16	16/4	16	8/4	AMP, AMC, CHL, FOX, STR, SUL, TET
AM21465	>32	0.12	>8	16	16	16/4	8	8/4	AMP, AMC, CHL, FOX, STR, SUL, TET
AM20428	>32	0.5	2	<0.25	0.5	0.5/4	0.5	0.12/4	(CHL), (FOX)
AM06960	>32	>4	8	8	0.5	0.25/4	1	0.12/4	AMP, AMC, (CHL), GEN, KAN, STR, SUL, SXT
AM08081	>32	>4	4	0.5	0.5	0.25/4	1	0.12/4	AMP, AMC, CHL, FOX, (GEN), KAN, STR, SUL, SXT
AM08208	>32	>4	2	<0.25	0.5	0.25/4	0.5	0.25/4	AMP, (AMC) CHL, GEN, KAN, STR, SUL, SXT, TET
AM09864	>32	>4	8	8	64	0.25/4	8	0.25/4	AMP, (CHL), (FOX), GEN, KAN
AM11007	>32	>4	4	0.5	1	0.5/4	1	0.5/4	AMP, AMC, CHL, (FOX), KAN, SUL
AM14058	>32	>4	>8	>64	64	64/4	128	>64/4	(AMI), AMP, AMC, CHL, FOX, KAN, SUL
AM16094	>32	>4	4	<0.25	0.5	0.25/4	1	0.25/4	AMP, (AMC), CHL, (FOX), (GEN), KAN, SUL, SXT
AM18622	>32	>4	8	1	2	0.5/4	4	0.5/4	AMP, AMC, CHL, KAN, STR, SUL, SXT
AM19422	>32	>4	4	<0.25	0.5	0.5/4	2	0.25/4	AMP, AMC, (GEN), KAN, STR, SUL, SXT
AM20227	>32	>4	2	<0.25	1	2/4	1	0.5/4	AMP, AMC, (CHL), (FOX), GEN, KAN, STR, SUL, SXT
AM20256	>32	>4	4	<0.25	0.5	0.5/4	1	0.25/4	AMP, (AMC), (CHL), (GEN), KAN, SUL, SXT
AM02544	256	0.25	>16	64	128	0.5/4	32	0.12/4	AMP, (AMC), KAN, STR, SUL, TET
AM08739	>32	0.25	>8	32	16	16/4	16	8/4	AMP, AMC, CHL, FOX, GEN, KAN, STR, SUL, TET
AM11682	>32	0.25	>8	16	16	8/4	16	8/4	AMP, AMC, FOX
AM14364	>32	0.25	>8	32	32	16/4	32	16/4	AMP, AMC, CHL, FOX, GEN, KAN, STR, SUL, TET
AM14807	>32	0.25	>8	16	32	16/4	16	32/4	AMP, AMC, CHL, FOX, STR, SUL, TET
AM20205	>32	0.25	2	<0.25	0.5	0.5/4	0.25	0.25/4	AMP, KAN, STR, SUL, TET
AM19537	>32	0.12	>8	16	16	16/4	8	8/4	AMP, AMC, CHL, FOX, (GEN), (KAN), STR, SUL TET

*NARMS, National Antimicrobial Resistance Monitoring System; NAL, nalidixic acid; CIP, ciprofloxacin; XNL, ceftiofur; CRO, ceftriaxone; TAZ, ceftazidime; TAZ/CLAV, ceftazidime/clavulanate; FOT, cefotaxime, FOT/CLAV, cefotaxime/clavulanate; AMP, ampicillin; AMC, amoxicillin/clavulanate; CHL, chloramphenicol; FOX, cefoxitin; KAN, kanamycin; STR, streptomycin; SUL, sulfamethoxazole or sulfisoxazole; SXT, trimethoprim/sulfamethoxazole; TET, tetracycline; AMI, amikacin; GEN, gentamicin. †Drugs in parentheses had intermediate results.

The mechanisms responsible for resistance and decreased susceptibility are shown in Table 4. Some mechanisms for some of the isolates are presented elsewhere (*3*,*17*,*18*). At least 1 *gyrA* mutation was found in 26 of 27 isolates. A *gyrA* mutation at codon 83 only was found for 11 isolates; a mutation at codon 87 only was found for 3; mutations at both codons were found for 12. No functional mutations were detected in *gyrB* or *parE* genes. All Senftenberg isolates had *parC* mutations (S80I and T57S), and 6 other isolates had the T57S mutation. In addition to the T57S mutation in *parC*, the Mbandaka isolate contained a plasmid-mediated *qnrB2* gene and has been described (*13*). Four isolates contained *aac(6′)-Ib*, but none contained the ciprofloxacin-modifying *aac(6′)-Ib-cr* variant.

**Table 4:** Resistance mechanisms among NARMS non-Typhi *Salmonella* with decreased susceptibility to quinolones and extended-spectrum cephalosporins, United States, 1996–2004*

Isolate	*gyrA* codon 83 change	*gyrA* codon 87 change	*parC* codon 57 change	*parC* codon 80 change	β-Lactamase isoelectric points	β-Lactamase genes
AM18280	S83Y	WT	T57S	WT	>8.4	*bla*_CMY-2_
AM09124	S83F	WT	WT	WT	ND	ND
AM15266	WT	D87Y	WT	WT	ND	ND
AM12389	S83Y	WT	WT	WT	ND	ND
AM15010	WT	WT	T57S	WT	7.0, 7.6†	*bla*_SHV-30_
AM03005	S83Y	D87G	T57S	WT	ND	ND
AM15201	S83F	WT	T57S	WT	>8.4	*bla*_CMY-2_
AM21465	S83F	WT	T57S	WT	>8.4	*bla*_CMY-2_
AM20428	S83F	WT	WT	WT	5.4	*bla*_TEM-1b_
AM06960	S83Y	D87G	T57S	S80I	7.4	*bla*_OXA-1_
AM08081	S83Y	D87G	T57S	S80I	7.4	*bla*_OXA-1_
AM08208	S83Y	D87G	T57S	S80I	7.4	*bla*_OXA-1_
AM09864	S83Y	D87G	T57S	S80I	5.3, 6.9, 8.0	*bla*_TEM-1_, *bla*_OXA-9_, *bla*_SHV-12_
AM11007	S83Y	D87G	T57S	S80I	7.4	*bla*_OXA-1_
AM14058	S83Y	D87G	T57S	S80I	5.4, >8.4	*bla*_TEM-1b_, *bla*_CMY-23_
AM16094	S83Y	D87G	T57S	S80I	7.4	*bla*_OXA-1_
AM18622	S83Y	D87G	T57S	S80I	7.4, 7.8	*bla*_OXA-1_
AM19422	S83Y	D87G	T57S	S80I	7.4	*bla*_OXA-1_
AM20227	S83Y	D87G	T57S	S80I	7.4	*bla*_OXA-1_
AM20256	S83Y	D87G	T57S	S80I	7.4	*bla*_OXA-1_
AM02544	WT	D87N	WT	WT	5.4, 8.0	*bla*_TEM-1_, *bla*_OXA-1_, *bla*_SHV-12_
AM08739	S83Y	WT	WT	WT	5.4, >8.4	*bla*_TEM-1b_, *bla*_CMY-2_
AM11682	S83F	WT	WT	WT	>8.4	*bla*_CMY-2_
AM14364	S83Y	WT	WT	WT	5.4, >8.4	*bla*_TEM-1b_, *bla*_CMY-2_
AM14807	S83Y	WT	WT	WT	>8.4	*bla*_CMY-2_
AM20205	WT	D87N	WT	WT	5.4	*bla*_TEM-1b_
AM19537	S83Y	WT	T57S	WT	>8.4	*bla*_CMY-2_

*NARMS, National Antimicrobial Resistance Monitoring System; WT, wild type; ND, not determined. †Gene responsible not yet identified.

Nine AmpC phenotype isolates produced β-lactamase with a pI >8.4 (Table 4); 8 contained *bla*_CMY-2_, but the Senftenberg strain contained a *bla*_CMY-23_ gene (GenBank accession no. DQ463751) identical to that found in an *E. coli* isolate (GenBank accession no. DQ438952). This gene differs from *bla*_CMY-2_ by 1 amino acid. Three of the *bla*_CMY_-positive isolates, including the strain positive for *bla*_CMY-23_, also contained *bla*_TEM-1b_. The Mbandaka isolate was positive for *bla*_SHV-30_ with pI 7.0 (*33*) and also produced an enzyme with a pI 7.6, the nature of which is still under study. Two isolates (1 Senftenberg and 1 Typhimurium) contained *bla*_SHV-12_, and both also contained *bla*_OXA_ and *bla*_TEM-1_ genes. Of the 11 Senftenberg isolates, 10 contained *bla*_OXA-1_ (n = 9) or *bla*_OXA-9_ (n = 1). No isolates contained *bla*_CTX-M_ genes.

## Discussion

Fluoroquinolone and extended-spectrum cephalosporin coresistance is rare; however, the appearance of this phenotype in 2 commonly isolated serotypes from humans (Typhimurium and Newport) is concerning. Sporadic infections are alarming, but if clonal expansion of an isolate with this phenotype were to take place, as occurred with *Salmonella* ser. Typhimurium DT104 and Newport-MDRAmpC, the clinical consequences could be dramatic. Statistically significant increases in resistance to nalidixic acid (odds ratio [OR] 6.7, 95% confidence interval [CI] CI 2.6–17.7) and ceftiofur (OR 43.2, 95% CI 10.5–177.4) have been documented among non-Typhi *Salmonella* of human origin submitted to NARMS during 1996–2003 (*4*). Of 202 nalidixic acid–resistant non-Typhi *Salmonella* collected by NARMS during 1996–2003, most were ser. Enteritidis (31%) or Typhimurium (10%). Most of the 324 ceftiofur-resistant non-Typhi *Salmonella* collected by NARMS during the same time period were ser. Newport (56%) or Typhimurium (23%). A slightly broader geographic representation can be found in the SENTRY surveillance project, which analyzed 786 *Salmonella* isolates (blood and stool) from medical facilities in Latin America and North America (including Canada) during 2001–2003 (*8*). Of these, 11% were resistant to nalidixic acid, and 2% exhibited decreased susceptibility to ceftazidime, ceftriaxone, or aztreonam.

Extended-spectrum cephalosporin-resistant Newport and Typhimurium isolates are typically obtained from community-acquired infections. Newport-MDRAmpC infections have been associated with consumption of contaminated beef and unpasteurized dairy products (*34*). *Salmonella* containing *bla*_CMY_ genes have been isolated from ground chicken (Typhimurium DT208), turkey (Agona), and beef (Agona) purchased from retail outlets in the Washington DC area (*35*). In addition, cattle, chickens, turkeys, pigs, horses, and dogs have all been sources of *bla*_CMY_-containing *Salmonella,* including common serotypes such as Typhimurium, Newport, and Heidelberg (*26*,*36*,*37*). Decreased susceptibility to fluoroquinolones among *Salmonella* serotypes that typically carry *bla*_CMY_ genes warrants exploration of factors that could select for decreased susceptibility to fluoroquinolones in animal reservoirs and in the human host.

PFGE showed diversity within some serotypes and indistinguishable strains within others. PFGE diversity among 2 serotypes commonly associated with extended-spectrum cephalosporin resistance (Newport and Typhimurium) is not surprising, given that CMY-producing strains have been seen at least since the late 1990s. Isolates of ser. Enteritidis are highly clonal; therefore, PFGE-indistinguishable patterns among isolates with no apparent epidemiologic link are not unusual. All PFGE-indistinguishable Senftenberg isolates from group 1 were isolated in the same state. Results for the Florida Senftenberg isolates are described elsewhere (*16*,*18*).

*Salmonella* ser. Senftenberg exhibiting decreased susceptibility to fluoroquinolones has been associated with nosocomial infections in healthcare facilities in the United States (*18*). All 11 isolates contained identical *gyrA* mutations (S83Y and D87G) and *parC* mutations (T57S and S80I). These *parC* mutations have been identified in several *Salmonella* serotypes including Senftenberg (*38*). Ten Senftenberg isolates included in this study contained *bla*_OXA_ genes; the *bla*_OXA_-negative Senftenberg strain contained a *bla*_CMY-23_ mechanism of extended-spectrum cephalosporin resistance. Acquisition of a *bla*_CMY_ gene by a traditionally nalidixic acid–resistant serotype warrants further epidemiologic and laboratory investigation. The *bla*_OXA-1_ gene has been identified in *Salmonella* ser. Typhimurium and is reported to be carried by an integron (*39*); *bla*_OXA-9_ has been associated with Tn1331 (*40*).

The epidemiology of *Salmonella* with decreased susceptibility to fluoroquinolones is relatively well characterized, as is that of *Salmonella* with *bla*_CMY_-mediated extended-spectrum cephalosporin resistance. Conversely, little is known about the events leading to quinolone and extended-spectrum cephalosporin coresistance and the epidemiology of these infections in humans. Patients with *Salmonella* infections who exhibit decreased susceptibility to both antimicrobial drug classes should be interviewed to determine risk factors and the effects of antimicrobial drugs and other potential selective factors on this phenomenon.

## References

[1] Hohmann EL. Nontyphoidal salmonellosis. Clin Infect Dis. 2001;32:263–9. 10.1086/318457 11170916

[2] Centers for Disease Control and Prevention. NARMS human isolates final report, 2002. 2005 [cited 2006 Nov 10]. Available from http://www.cdc.gov/narms/annual/2002/2002annualreportfinal.pdf

[3] Stevenson JE, Gay K, Barrett TJ, Medalla F, Chiller TM, Angulo FJ. Increase in nalidixic acid resistance among non-Typhi *Salmonella enterica* isolates in the United States from 1996–2003. Antimicrob Agents Chemother. 2007;51:195–7. 10.1128/AAC.00222-06 17088493 PMC1797669

[4] Centers for Disease Control and Prevention. NARMS human isolates final report, 2003. 2006 [cited 2006 Nov 11]. Available from http://www.cdc.gov/narms/annual/2003/NARMS2003AnnualReport.pdf

[5] Hakanen AJ, Lindgren M, Huovinen P, Jalava J, Siitonen A, Kotilainen P. New quinolone resistance phenomenon in *Salmonella enterica*: nalidixic acid-susceptible isolates with reduced fluoroquinolone susceptibility. J Clin Microbiol. 2005;43:5775–8. 10.1128/JCM.43.11.5775-5778.2005 16272517 PMC1287832

[6] Dunne EF, Fey PD, Kludt P, Reporter R, Mostashari F, Shillam P, Emergence of domestically acquired ceftriaxone-resistant *Salmonella* infections associated with AmpC β-lactamase. JAMA. 2000;284:3151–6. 10.1001/jama.284.24.3151 11135779

[7] Carattoli A, Tosini F, Giles WP, Rupp ME, Hinrichs SH, Angulo FJ, Characterization of plasmids carrying CMY-2 from expanded-spectrum cephalosporin-resistant *Salmonella* strains isolated in the United States between 1996 and 1998. Antimicrob Agents Chemother. 2002;46:1269–72. 10.1128/AAC.46.5.1269-1272.2002 11959555 PMC127137

[8] Biedenbach DJ, Toleman M, Walsh TR, Jones RN. Analysis of *Salmonella* spp. with resistance to extended-spectrum cephalosporins and fluoroquinolones isolated in North America and Latin America: report from the SENTRY Antimicrobial Surveillance Program (1997–2004). Diagn Microbiol Infect Dis. 2006;54:13–21. 10.1016/j.diagmicrobio.2005.06.013 16290025

[9] Boonmar S, Bangtrakulnonth A, Pornruangwong S, Samosornsuk S, Kaneko K, Ogawa M. Significant increase in antibiotic resistance of *Salmonella* isolates from human beings and chicken meat in Thailand. Vet Microbiol. 1998;62:73–80. 10.1016/S0378-1135(98)00194-1 9659693

[10] Threlfall EJ, Skinner JA, Graham A, Ward LR, Smith HR. Resistance to ceftriaxone and cefotaxime in non-typhoidal *Salmonella enterica* in England and Wales, 1998–99. J Antimicrob Chemother. 2000;46:860–2. 10.1093/jac/46.5.860 11062222

[11] Bertrand S, Weill FX, Cloeckaert A, Vrints M, Mairiaux E, Praud K, Clonal emergence of extended-spectrum beta-lactamase (CTX-M-2)–producing *Salmonella enterica* serovar Virchow isolates with reduced susceptibilities to ciprofloxacin among poultry and humans in Belgium and France (2000 to 2003). J Clin Microbiol. 2006;44:2897–903. 10.1128/JCM.02549-05 16891509 PMC1594617

[12] Prabha Adhikari MR, Baliga S. Ciprofloxacin-resistant typhoid with incomplete response to cefotaxime. J Assoc Physicians India. 2002;50:428–9. 11924574

[13] Gay K, Robicsek A, Strahilevitz J, Park CH, Jacoby G, Barrett TJ, Plasmid-mediated quinolone resistance in non-Typhi serotypes of *Salmonella enterica.* Clin Infect Dis. 2006;43:297–304. 10.1086/505397 16804843

[14] Yan JJ, Chiou CS, Lauderdale TL, Tsai SH, Wu JJ. Cephalosporin and ciprofloxacin resistance in *Salmonella*, Taiwan. Emerg Infect Dis. 2005;11:947–50. 15963294 10.3201/eid1106.041153 PMC3367602

[15] Zhao S, Fedorka-Cray PJ, Friedman S, McDermott PF, Walker RD, Qaiyumi S, Characterization of *Salmonella* Typhimurium of animal origin obtained from the National Antimicrobial Resistance Monitoring System. Foodborne Pathog Dis. 2005;2:169–81. 10.1089/fpd.2005.2.169 15992312

[16] Stamey K, Rossiter S, Kubota K, VanDuyne S, Blackmore C, Farah R, PFGE patterns of multidrug-resistant *Salmonella* serotype Senftenberg isolated from patients in a Florida hospital nosocomial transmission. Proceedings of the 101st General Meeting Abstracts; 2001 May 20–24; Orlando (FL). Washington: American Society for Microbiology; 2001.

[17] Zuppardo A, Fiorella PD, Baker R, Farah R, Sieffert D, Hale Y. Genetic profiles of multidrug resistant strains of *Salmonella* Senftenberg from a nosocomial Florida outbreak. Proceedings of the 102nd General Meeting Abstracts; 2002 May 19–23; Salt Lake City (UT). Washington; American Society for Microbiology; 2002.

[18] Kay RS, Vandevelde AG, Fiorella PD, Crouse R, Blackmore C, Sanderson R, Outbreak of healthcare-associated infection and colonization with multidrug-resistant *Salmonella enterica* serovar Senftenberg in Florida. Infect Control Hosp Epidemiol. 2007;28:805–11. 10.1086/518458 17564982

[19] Popoff MY. Antigenic formulas of the *Salmonella* serovars. 8th ed. Paris: World Health Organization Collaborating Centre for Reference and Research on *Salmonella*, Institute Pasteur; 2001.

[20] Clinical Laboratory Standards Institute. Performance standards for antimicrobial susceptibility testing; Sixteenth Informational Supplement (M100-S16). Wayne (PA): The Institute; 2006.

[21] Rasheed JK, Anderson GJ, Yigit H, Queenan AM, Domenech-Sanchez A, Swenson JM, Characterization of the extended-spectrum beta-lactamase reference strain, *Klebsiella pneumoniae* K6 (ATCC 700603), which produces the novel enzyme SHV-18. Antimicrob Agents Chemother. 2000;44:2382–8. 10.1128/AAC.44.9.2382-2388.2000 10952583 PMC90073

[22] Jacoby GA, Chow N, Waites KB. Prevalence of plasmid-mediated quinolone resistance. Antimicrob Agents Chemother. 2003;47:559–62. 10.1128/AAC.47.2.559-562.2003 12543659 PMC151764

[23] Jacoby GA, Walsh KE, Mills DM, Walker VJ, Oh H, Robicsek A, qnrB, another plasmid-mediated gene for quinolone resistance. Antimicrob Agents Chemother. 2006;50:1178–82. 10.1128/AAC.50.4.1178-1182.2006 16569827 PMC1426915

[24] Park CH, Robicsek A, Jacoby GA, Sahm D, Hooper DC. Prevalence in the United States of *aac(6')-Ib-cr* encoding a ciprofloxacin-modifying enzyme. Antimicrob Agents Chemother. 2006;50:3953–5. 10.1128/AAC.00915-06 16954321 PMC1635235

[25] Winokur PL, Vonstein DL, Hoffman LJ, Uhlenhopp EK, Doern GV. Evidence for transfer of CMY-2 AmpC beta-lactamase plasmids between *Escherichia coli* and *Salmonella* isolates from food animals and humans. Antimicrob Agents Chemother. 2001;45:2716–22. 10.1128/AAC.45.10.2716-2722.2001 11557460 PMC90722

[26] Chen S, Zhao S, White DG, Schroeder CM, Lu R, Yang H, Characterization of multiple-antimicrobial-resistant *Salmonella* serovars isolated from retail meats. Appl Environ Microbiol. 2004;70:1–7. 10.1128/AEM.70.1.1-7.2004 14711619 PMC321239

[27] Brinas L, Zarazaga M, Saenz Y, Ruiz-Larrea F, Torres C. Beta-lactamases in ampicillin-resistant *Escherichia coli* isolates from foods, humans, and healthy animals. Antimicrob Agents Chemother. 2002;46:3156–63. 10.1128/AAC.46.10.3156-3163.2002 12234838 PMC128764

[28] Rasheed JK, Jay C, Metchock B, Berkowitz F, Weigel L, Crellin J, Evolution of extended-spectrum beta-lactam resistance (SHV-8) in a strain of *Escherichia coli* during multiple episodes of bacteremia. Antimicrob Agents Chemother. 1997;41:647–53. 9056008 10.1128/aac.41.3.647 PMC163766

[29] Yagi T, Kurokawa H, Shibata N, Shibayama K, Arakawa Y. A preliminary survey of extended-spectrum beta-lactamases (ESBLs) in clinical isolates of *Klebsiella pneumoniae* and *Escherichia coli* in Japan. FEMS Microbiol Lett. 2000;184:53–6. 10689165 10.1111/j.1574-6968.2000.tb08989.x

[30] Woodford N, Fagan EJ, Ellington MJ. Multiplex PCR for rapid detection of genes encoding CTX-M extended-spectrum β-lactamases. J Antimicrob Chemother. 2006;57:154–5. 10.1093/jac/dki412 16284100

[31] Eaves DJ, Randall L, Gray DT, Buckley A, Woodward MJ, White AP, Prevalence of mutations within the quinolone resistance-determining region of *gyrA, gyrB, parC*, and *parE* and association with antibiotic resistance in quinolone-resistant *Salmonella enterica.* Antimicrob Agents Chemother. 2004;48:4012–5. 10.1128/AAC.48.10.4012-4015.2004 15388468 PMC521866

[32] Ribot EM, Fair MA, Gautom R, Cameron DN, Hunter SB, Swaminathan B, Standardization of pulsed-field gel electrophoresis protocols for the subtyping of *Escherichia coli* O157:H7, *Salmonella*, and *Shigella* for PulseNet. Foodborne Pathog Dis. 2006;3:59–67. 10.1089/fpd.2006.3.59 16602980

[33] Szabo D, Melan MA, Hujer AM, Bonomo RA, Hujer KM, Bethel CR, Molecular analysis of the simultaneous production of two SHV-type extended-spectrum beta-lactamases in a clinical isolate of *Enterobacter cloacae* by using single-nucleotide polymorphism genotyping. Antimicrob Agents Chemother. 2005;49:4716–20. 10.1128/AAC.49.11.4716-4720.2005 16251316 PMC1280125

[34] Varma JK, Marcus R, Stenzel SA, Hanna SS, Gettner S, Anderson BJ, Highly resistant *Salmonella* Newport-MDRAmpC transmitted through the domestic US food supply: a FoodNet case-control study of sporadic *Salmonella* Newport infections, 2002–2003. J Infect Dis. 2006;194:222–30. 10.1086/505084 16779729

[35] White DG, Zhao S, Sudler R, Ayers S, Friedman S, Chen S, The isolation of antibiotic-resistant *Salmonella* from retail ground meats. N Engl J Med. 2001;345:1147–54. 10.1056/NEJMoa010315 11642230

[36] Rankin SC, Aceto H, Cassidy J, Holt J, Young S, Love B, Molecular characterization of cephalosporin-resistant *Salmonella enterica* serotype Newport isolates from animals in Pennsylvania. J Clin Microbiol. 2002;40:4679–84. 10.1128/JCM.40.12.4679-4684.2002 12454172 PMC154621

[37] Winokur PL, Brueggemann A, DeSalvo DL, Hoffmann L, Apley MD, Uhlenhopp EK, Animal and human multidrug-resistant, cephalosporin-resistant *Salmonella* isolates expressing a plasmid-mediated CMY-2 AmpC beta-lactamase. Antimicrob Agents Chemother. 2000;44:2777–83. 10.1128/AAC.44.10.2777-2783.2000 10991860 PMC90151

[38] Hopkins KL, Arnold C, Threlfall EJ. Rapid detection of *gyrA* and *parC* mutations in quinolone-resistant *Salmonella enterica* using pyrosequencing technology. J Microbiol Methods. 2007;68:163–71. 10.1016/j.mimet.2006.07.006 16934351

[39] Yamamoto T, Tanaka M, Nohara C, Fukunaga Y, Yamagishi S. Transposition of the oxacillin-hydrolyzing penicillinase gene. J Bacteriol. 1981;145:808–13. 6257651 10.1128/jb.145.2.808-813.1981 PMC217183

[40] Tolmasky ME, Crosa JH. Genetic organization of antibiotic resistance genes (*aac(6')-Ib, aadA*, and *oxa9*) in the multiresistance transposon Tn1331. Plasmid. 1993;29:31–40. 10.1006/plas.1993.1004 8382826

